# Author Correction: Octet lattice-based plate for elastic wave control

**DOI:** 10.1038/s41598-022-08219-8

**Published:** 2022-03-09

**Authors:** Giulia Aguzzi, Constantinos Kanellopoulos, Richard Wiltshaw, Richard V. Craster, Eleni N. Chatzi, Andrea Colombi

**Affiliations:** 1 Department of Civil, Environmental and Geomatic Engineering, ETH Zürich, 8093 Zürich, Switzerland; 2 Department of Mathematics, Imperial College London, London, SW7 2AZ UK; 3 Department of Mechanical Engineering, Imperial College London, London, SW7 2AZ UK; 4 Abraham de Moivre‑CNRS IRL, Imperial College London, London, SW7 2AZ UK

Correction to: *Scientific Reports* 10.1038/s41598-022-04900-0, published online 20 January 2022

The Acknowledgements section in the original version of this Article was incomplete.

“GA and AC were supported by the H2020 FETOpen project BOHEME under Grant agreement No. 863179 and by the Ambizione Fellowship PZ00P2-174009. CK was supported by the H2020 “INSPIRE” EU program under Grant agreement No. 813424. RW thanks the UK EPSRC for their support through Grant EP/L016230/1.”

now reads:

“GA and AC were supported by the H2020 FETOpen project BOHEME under Grant agreement No. 863179 and by the Swiss National Science Foundation Ambizione Fellowship PZ00P2-174009. CK was supported by the H2020 “INSPIRE” EU program under Grant agreement No. 813424. RW thanks the UK EPSRC for their support through Grant EP/L016230/1."

The original Article has been corrected.

