# Supplementary material for: Octet lattice-based plate for elastic wave control
Source: Sci Rep. 2022 Jan 20;12:1088. doi: 10.1038/s41598-022-04900-0 (PMC8776834; doi:10.1038/s41598-022-04900-0)
Supplement: Supplementary file 1 — Supplementary Information 1. [file 41598_2022_4900_MOESM1_ESM.zip › Supplementary_Material/Supplementary_Material.pdf]

Supplementary Material to Octet lattice-based plate for elastic wave control

Giulia Aguzzi*, Constantinos Kanellopoulos, Richard Wiltshaw, Richard V. Craster, Eleni N. Chatzi and Andrea Colombi

** Corresponding Author. Email: aguzzi@ibk.baug.ethz.ch*

SM1: Complete band structure

To demonstrate the effectiveness of the octet lattice dynamics regardless of the considered direction and motivate our choice of showing the band structure only along Γ-X in the main manuscript, we have here calculated and reported the full dispersion relation of the standard octet cell illustrated in Fig. 1a. Following the procedure outlined in Methods for the Bloch analysis, we have solved the eigenvalue problem for the wavevector **k** sweeping on the entire Irreducible Brillouin Zone (IBZ) using COMSOL Multiphysics.

The dispersion relation in Fig. SM1 clearly shows that the bandgap (gray region) extends over the full IBZ. Even when examining the complete band structure, this attenuation zone is inferiorly and superiorly delimited by modes occurring along Γ-X with out-of-plane polarization, as reported in Fig. SM1*b*. While the lower bound mode remains consistent across the whole wavelength spectrum (blue and green circles in Fig. SM1*b* and *c*), the out-of-plane mode in direction M-Γ occurs at a higher frequency, respectively 5.18 kHz instead of 5.08 kHz (right insets in Fig. SM1*b* and *c* and further highlighted by the green and blue dotted lines in *a*).

In addition, based on this full dispersion relation, we expect the same results outlined in the manuscript to be obtained when considering different directions, e.g., M-Γ (45° between *x* and *y* in the physical space).

Based on these remarks, we can conclude that the Γ-X portion of the band structure is crucial in the analysis of the dynamic behavior of the octet cell and captures all the relevant features to fulfill the objectives of this study.

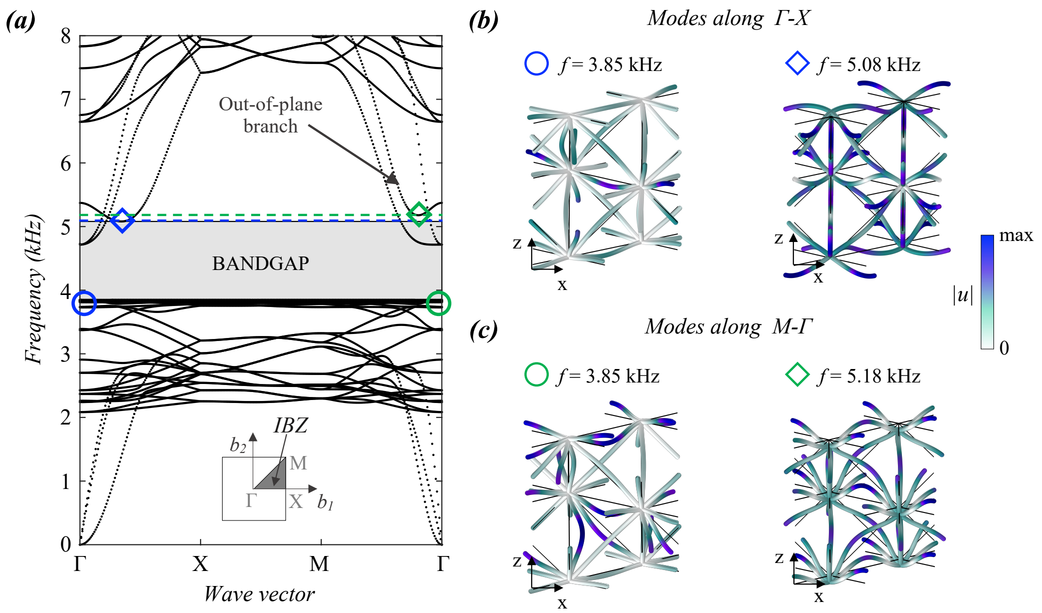

Figure SM1: Full dispersion relation. (a) Complete band structure calculated via Bloch analysis in COMSOL Multiphysics along the edges of the Irreducible Brillouin Zone Γ-X-M-Γ (gray triangle in the inset), where $\mathbf{b_1}$ and $\mathbf{b_2}$ are the reciprocal lattice vectors. The pass bands in the Γ-X direction correspond to the dispersion curves illustrated in red in Fig. 2*a*. (b) Opening and closing modes of the bandgap along Γ-X, the same as those reported in Fig. 2*b*. (c) Opening and closing modes of the bandgap along M-Γ.

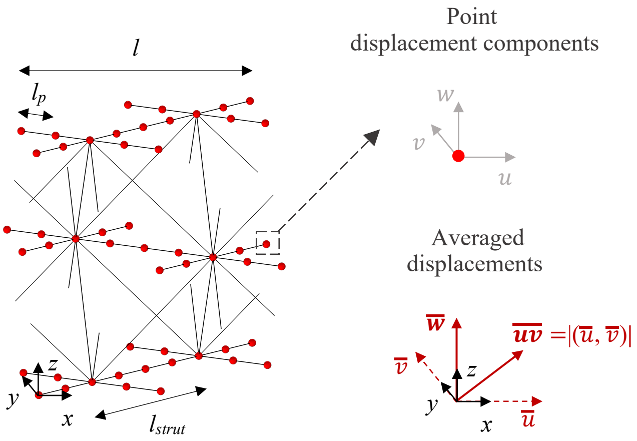

Figure SM2: Schematic considered in the computation of the plate macroscale dominant polarization. The points where the displacement components (*u*, *v* and *w*) have been extracted are highlighted in red.

SM2: Plate motion and mode shapes

A set of deformations emerges when considering the wave modes propagating in the octet-based plate, identified by the dispersion relation in Fig. 2*a*, which is worth analyzing in more detail. In order to comprehensively understand the dynamics of this structure, we first need to distinguish between motions occurring at two different scales. The *microscale* involves deformations localized in the struts, which include their compressional, flexural and, at higher frequencies than those presented in this study, torsional motion. Since the structure contains struts whose neutral axes are not all aligned, their intersection at the nodes ensures a coupling of these different types of motion for conserved quantities (forces, moments, torques, etc.). These motions combine at the *macroscopic* scale, leading the plate assembly to deform either orthogonally (along *z*) or in parallel (*xy*) to the mid-plane.

For this analysis we will focus primarily on the second case.

Figure SM3*a* depicts the octet band structure, obtained via Bloch analysis in COMSOL (see Methods), with the corresponding polarization highlighted at each point. Each frequency-wavenumber pair is enriched with a different color representing, respectively, out-of-plane (pink) or in-plane (light blue) deformations of the plate at the macroscale level. In order to compute these polarizations, we have extracted the three displacement components (*u*, *v* and *w*) in each of the red dots of the fundamental unit cell illustrated in Fig. SM2 (same as the one in Fig. 1*a*). We have considered only points along the struts parallel to the mid-plane of the plate (surface *xy*), since the other members are tilted with respect to this surface and complicate the separation of the directions. A distance l_p, equal to $l_{strut}/8$, is assumed between each point to prevent them from matching the nodes of the strut modes, as these undergo zero displacement and provide irrelevant information. For each pair of values in the dispersion relation (*k*, *f*), we have then averaged every displacement component separately over all the points extracted and identified the correspondent mean values $\overline{u}$, $\overline{v}$ and $\overline{w}$. Since both $\overline{u}$ and $\overline{v}$ represent displacements in the plane parallel to the mid-surface, we have calculated their modulus, $\overline{uv} = |(\overline{u}, \overline{v})|$, in the bottom-right inset of Fig. SM2, hence obtained two final distinct components, one in-plane $\overline{uv}$ and the other out-of-plane $\overline{w}$. Finally, we have determined the type of motion of the plate by computing the ratio between these two components, $\overline{w}/\overline{uv}$, and further validated this procedure by comparing the estimated polarizations with the correspondent mode shapes in COMSOL.

The complexity in discerning the in-plane from the out-of-plane macro-polarizations in Fig. SM3*a* is closely related to the range of frequencies analyzed.

Between 0 and 2 kHz, as explained in the main text, the octet-based plate has a subwavelength behavior and resembles an equivalent homogeneous continuum. Therefore, it is characterized by three modes with clear polarizations, one out-of-plane (pink) and two in-plane (light blue), respectively. In this figure, the wavevector sweeping the Γ-X direction corresponds to waves propagating along *x*. The out-of-plane polarization emerges as an almost rigid *z*-wise translation of the octet cell (left inset in Fig. SM3*b*, 1st mode), while the same motion involves the *y*, and consequently *x*, direction for the in-plane mode (2nd mode).

Similarly, at frequencies above 4 kHz the polarization of the modes can be clearly distinguished, as evidenced by the colors in Fig. SM3*a*. Owing to the higher frequencies and shorter wavelengths, these modes localize in the struts, which exhibit flexural deformations combined with rotations that also involve the nodes (see Fig. SM3*d*). These rotations occur either out-of-plane (see mode 44th), or in-plane (see mode 46th), but are always consistent with the overall polarization of the plate.

Between 2 and 4 kHz, it is seemingly more complex to clearly separate the motion direction of the modes, as the waves interact with the intricate network of struts and activate a more sophisticated set of deformations. As reported in Fig. SM3*c*, some of these modal shapes show a combination of out-of-plane rotational motion of some

struts and in-plane bending of others, which make it difficult to identify a comprehensive polarization of the plate assembly. We can therefore conclude that, despite two dominant macro deformations of the plate being identified for most frequencies, it is not always possible to fully decouple these.

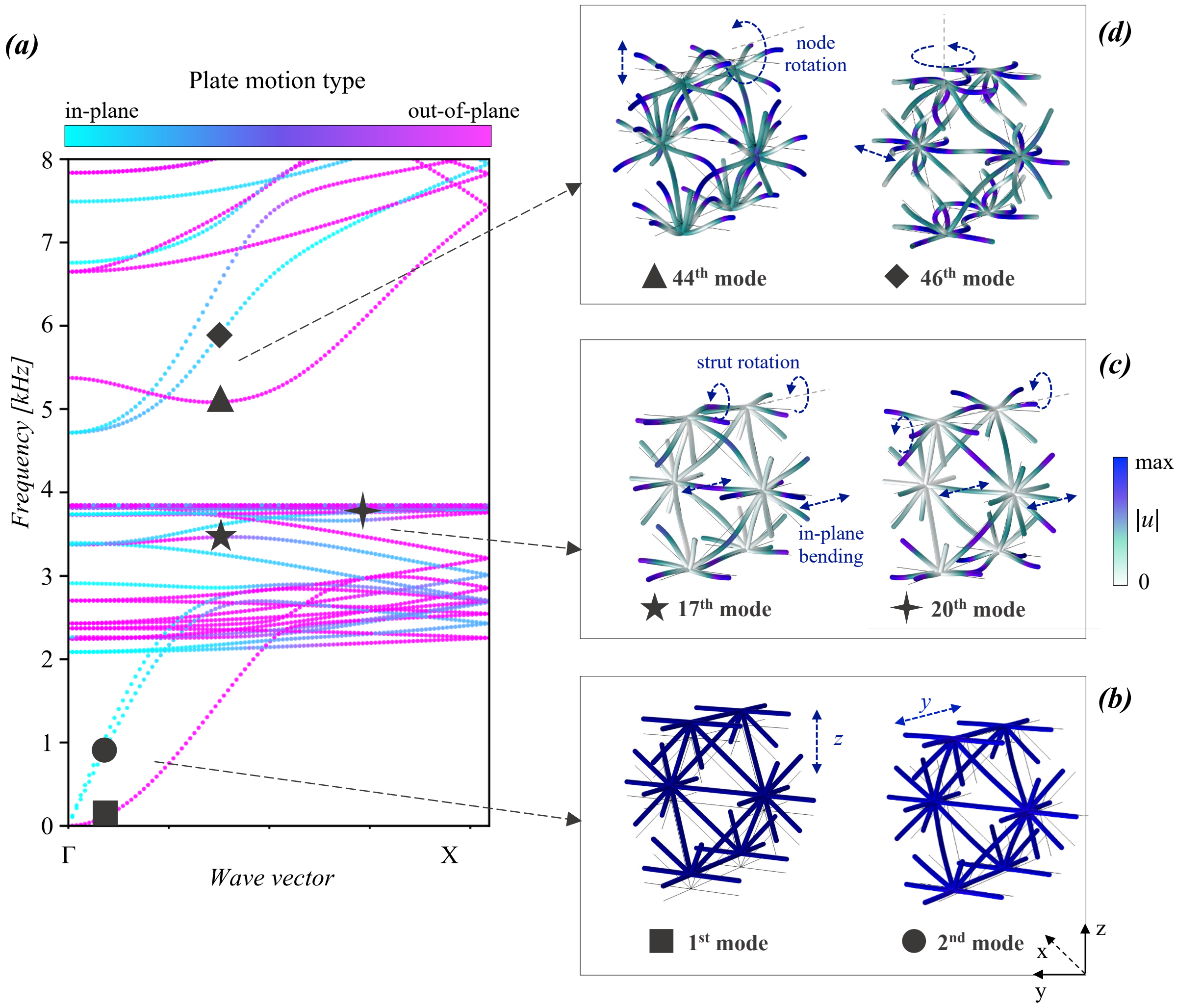

Figure SM3: Macroscopic motion polarization of the plate. (a) Band structure of the octet cell, where the motion polarization in each point of the dispersion curves is highlighted. (b), (c), (d) mode shapes in the three main frequency ranges of the dispersion relation. The animations of these modal shapes are additionally reported in the Supplementary videos.
